# Intersectional stigma and the arc of intranational migration: experiences of transgender adolescents and women who migrate within Peru

**DOI:** 10.1186/s12889-023-15985-1

**Published:** 2023-06-21

**Authors:** Amaya Perez-Brumer, Ximena Salazar, Aron Nunez-Curto, Lynne D’Amico, Rodrigo Aguayo-Romero, Sari L. Reisner, Alfonso Silva-Santisteban

**Affiliations:** 1 Division of Social and Behavioural Health, Dalla Lana School of Public Health, University of Toronto, Toronto, Canada; 2 Center for Interdisciplinary Research in Sexuality, AIDS and Society, Universidad Peruana Cayetano Heredia, Lima, Peru; 3 Knowledge-Shaping Solutions, LLC, Alexandria, USA; 4 Brigham and Women’s Hospital, Boston, MA USA; 5 Harvard Medical School, Boston, MA USA; 6 Harvard T.H. Chan School of Public Health, Boston, MA USA; 7 The Fenway Institute, Fenway Health, Boston, MA USA

**Keywords:** Transgender adolescents, Transgender women, Intranational migration, Intersectional stigma, Transphobia, Latin America

## Abstract

**Background:**

Migration is recognized as a key determinant of health. Yet, limited research addresses the arc of intranational migration and, even less, the experiences of transgender (trans) adolescents and women migrants and the associated health vulnerabilities. Using intersectional stigma as a theoretical frame, this study seeks to better understand the sexual health vulnerabilities and needs of trans women migrants in Peru.

**Methods:**

Between October and November 2016, in-depth interviews (n = 14) and two focus groups (n = 20) were conducted in Spanish with trans women in three Peruvian cities. To explore pre- and during migration experiences, focus groups were conducted in Pucallpa and Iquitos, key cities in the Amazon where trajectories often originate. To assess during migration and post-migration experiences, we conducted interviews in Pucallpa, Iquitos, and Lima to better understand processes of relocation. Audio files were transcribed verbatim and analysed via an immersion crystallization approach, an inductive and iterative process, using Dedoose (v.6.1.18).

**Results:**

Participants described migration as an arc and, thus, results are presented in three phases: pre-migration; during migration; and post-migration. Intersectional stigma was identified as a transversal theme throughout the three stages of migration. The pre-migration stage was characterized by poverty, transphobia, and violence frequently motivating the decision to migrate to a larger city. Exploitation was also described as pervasive during migration and in relocation. Many participants spoke of their introduction to sex work during migration, as key to economic earning and associated violence (police, clients).

**Conclusion:**

Findings advance understandings of intranational migration and forced displacement as key determinants of trans women’s health. Dimensions of violence at the intersection of classism and cisgenderism render trans women highly vulnerable at every step of their migratory journeys. Experiences of intranational mobility and relocation were described as uniquely tied to age, intersectional transphobic stigma, engagement in sex work, and multiple forms of violence, which impact and can magnify sexual health vulnerabilities for transgender women in Peru who migrated intranationally.

## Introduction

Migration, the temporary or permanent movement away from one’s place of residence to a place within or outside of state borders, has been identified as a key determinant of health [1]. Given the pervasiveness of transphobic violence globally, many transgender (trans) people migrate to urban centers and/or other countries to seek improved legal and social protections [2–5]. A host of social factors (e.g., limited legal protections, housing instability, economic precarity, informal labor) intersect to impact health across the migration trajectory, including, before, during, and after relocation [6]. It is critical to underscore migration as a process to better assess why experiences of migration among trans people are associated with increased health risks, including elevated HIV prevalence [7–10], and psychological distress [11, 12].

A recent scoping review highlights that the majority of health science literature on the relationship between migration, gender identity, and health has focused on the experiences of cross-border mobility among trans migrants who have relocated to the United States [5]. Yet, for many trans people, especially those in low- and middle-income country (LMIC) settings, the process of migration starts with intranational migration, meaning movement within a country, and at a young age. For trans migrants, health vulnerabilities and outcomes associated with intranational, and transnational migration may differ from those associated with international migration, given that transnational migration typically requires more social and financial resources [2, 13]. While intranational migrants may not experience vulnerabilities related to legal status or citizenship in the destination country, they may be more likely to experience housing precarity, impoverishment, and informal employment due to these socioeconomic differences [2, 14].

There is an urgent need to identify and understand the unique migration experiences and associations with health among transwomen who live in global regions where transphobia is heightened. Latin America is one of the deadliest regions for trans women. In 2022, the Trans Murder Monitoring observatory reported that of the murders of 327 transgender and gender-diverse people were recorded worldwide between the start of October 2021 and the end of September 2022, 95% were among trans women, and 68% of the murders, the most globally, took place in Latin America and the Caribbean [15]. In this context, trans women who engage in sex work, especially street-based sex work, often experience heightened stigma, discrimination, and violence from clients, law enforcement and medical professionals [3, 16–18]. Global scholarship has identified that anti-transgender and anti-immigration stigma often intersect and can independently and jointly drive increased exposure to physical and sexual violence; further, such experiences are associated with increased vulnerability to sexual health and mental health concerns [4, 5, 19].

Amid pervasive transphobia in Latin America, using intersectional stigma as a theoretical frame, this paper seeks to better understand the health vulnerabilities of trans women migrants in Peru. Intersectional stigma refers to the multiple, *interdependent* forms of stigma including symbolic, internalized, and enacted that operate across the micro, meso, and macro levels [20–22]. This is particularly important to consider when assessing how Peruvian trans women with multiple stigmatizing identities experience migration, as well as, how, multiple systems of oppression impact migration trajectories and are associated with health outcomes. For example, there are currently no laws that protect Peruvians from discrimination based on gender-identity [23]. Peruvian trans women experience extreme stigma and associated violence perpetrated by partners [24, 25], law enforcement [26–28], and health providers [29, 30]. Within Peru, trans women experience HIV-related stigma in social and medical environments as they are most affected by HIV, with a prevalence as high as 30% [31], a markedly higher prevalence than general population in Lima, which is between 0.2% and 0.3% [32]. Precarious labor and associated stigma must also be considered central to the living reality of many Peruvian trans women. Studies have reported that among trans women living in Lima 64% engage in sex work, and sex work is associated with migration from rural provinces to larger Peruvian cities [31, 33].

Contributing to this needed scholarship, we qualitatively examined the experiences of Peruvian trans adolescents and women who had embarked on migration trajectories within Peru. This paper addresses gaps in knowledge and understanding as to how numerous interrelated factors impact health vulnerabilities for trans women across the axes of intranational migration, socioeconomic status, age, and gender identity.

## Methods

Between October and November 2016, in-depth interviews (N= 14) and two focus group discussion (each had 10 participants, N = 20) were conducted with trans adolescents and women in three cities across Peru: Lima (the capital of Peru), and two cities located in the Amazonian region of Peru (Pucallpa and Iquitos). To explore trajectories of migration, focus groups were only conducted in Pucallpa and Iquitos, key cities in the Amazon, to assess pre– and during– migration experiences. We also conducted in-depth interviews in Pucallpa, Iquitos, and Lima (during– and post-migration) to better understand the full trajectory of the migration experience and processes of relocation.

City selection was based on formative work [23, 31, 33] and with guidance from Red Trans, a trans-led community organization primarily located in Lima but that has a network of affiliated grassroots organization by and for trans women across 18 of 24 regions in Peru. Lima is the capital of Peru and where approximately one-third of the national population reside. While transphobia is pervasive in Peru [23], there are greater employment opportunities, larger networks of trans women, and more access to gender-affirming medical care in Lima, making the city a key destination for many Peruvian trans women. Pucallpa and Iquitos are the largest cities in the Amazonian region of Peru, cities where intranational migration trajectories often originate [33].

### Participant eligibility and recruitment

Eligibility criteria included: being assigned male sex at birth and identifying on the transfeminine continuum (e.g., “woman”, “trans,” “transgender,” “travesti”), being ages 15 years or older, and having engaged in intranational migration. Purposive sampling [34] was implemented in collaboration with local recruiters from Red Trans. Participants under the age of 18 were eligible to participate in individual interviews and were currently living in Iquitos, Pucallpa, or Lima. Minor age participants were assented to participate in interviews and received additional counseling by study staff experienced in research involving minor age participants. Participants over the age of 18 were eligible to participate in individual interviews if they currently lived in Iquitos or Pucallpa, and/or had migrated to Lima from different areas of Peru. Recruitment was conducted through community leaders who were linked to Red Trans who supported in the initial identification of and contact with potential participants in each city (Pucallpa, Iquitos, and Lima).

### Data collection procedures

In-depth semi-structured interviews lasted between 20 and 30 min and explored themes related to gender identity, sex work, motivations for migration and rationale for anticipated destination city within Peru, processes of intranational migration, experiences after arriving in current city (Pucallpa, Iquitos, or Lima), and risks and support systems for trans women migrants. Six adolescent trans women and eight adult trans women completed in-depth interviews. Two focus groups (FGs) with transgender adolescents and women (n = 20) were conducted. The FGs were conducted in Pucallpa and Iquitos with 10 participants per group, lasting between 40 and 50 min. The groups covered topics related to the central theme of migrating as an adolescent: life prior to migrating, process of migrating, experiences after arriving in Lima, and future goals. Both in-depth interviews and FGs were recorded, all audio files were de-identified and transcribed in Spanish, and then translated to English.

All methods were carried out in accordance with relevant guidelines and regulations (e.g., Declaration of Helsinki) and the study protocol and instruments were approved by the Institutional Ethics Committee of the Universidad Peruana Cayetano Heredia (protocol #102991). All participants over 18 years of age completed a written informed consent prior to participation. For minor-aged participants, written informed consent was sought from their legal guardians. However, all trans youth in our sample were not in contact with their legal parents, and thus, minors provided informed assent. A de-identified random number was assigned to each audio file when transcribing and archiving the audio recordings. Participants were paid 30 Peruvian Soles (approximately $9.50 USD) for their time and were provided snacks.

### Data analysis

Given the numerous interrelated factors associated with the health vulnerabilities of our study population, we engaged with intersectional stigma as our guiding theoretical frame during analysis. This theoretical framing guided us to better understand how various intersecting forms of stigma and discrimination shape HIV vulnerabilities among adolescent and adult trans women who migrated intranationally to a medium/large-sized city in Peru. Research team members, who were native Spanish speakers, conducted the interviews and FGs. Both interviews and FGs were audio recorded and transcribed verbatim. Data analysis was guided by Immersion Crystallization, an inductive and iterative process that includes reading the raw data closely and engaging in an inductive, iterative process for identifying themes, categories, and patterns in the data to inform codebook creation [35]. Based on this close reading, a codebook was created, and transcripts were reviewed line-by-line to attribute codes. Dedoose qualitative data analysis software (2014, Los Angeles, CA: SocioCultural Research Consultants, LLC) was used to conduct all analyses. To increase rigor, the same team members involved in the interview and FG process were responsible for creating the codebook and systematically coding the data across sources. Data was triangulated across sources through comparing codes and creating memos that expanded on the relationships within the coded text [36]. The research team discussed and resolved any coding differences that arose at team meetings. Pseudonyms were employed in all reported data to protect the identities of participants.

## Results

Underscoring that migration is a process and to capture migration trajectories, results are presented in three phases: pre-migration; during migration; and post-migration. All participants had initiated their international migration process, most prior to 18 years of age. However, some were actively in the pre-and during-migration process, while others recalled their experiences of migration post-relocation. Intersectional stigma was identified as a transversal theme throughout the stages of migration, reflecting the complexity and for many, the ongoing characteristic of migration trajectories. Illustrative quotes for the three phases of migration are presented in Tables 1, 2 and 3. Among FGs in Pucallpa and Iquitos (N = 20 total participants), no demographic was collected. In the 14 interviews, the mean age was 20 years, ranging from 15 to 30 years old.

**Table 1:** Illustrative quotes of motivations for migration at pre-migration phase

	Illustrative quotes
1	“There was no financial support where I came from, I felt alone… economically I was bad, bad place” – Interview participant, 36 years old, Lima
2	“I didn’t finish high school, I just did one year. I did not want to go and there was no money. I had to go” – Interview participant, 20 years old, Pucallpa
3	“When I was 6 years old my mother started to leave me. When I was 7 years old, my mother left me to go on a trip and never came back.” – Interview participant, 17 years old, Pucallpa
4	“They [family] did not want to accept me because I wanted to be a trans girl” – Focus group participant, Pucallpa
5	“[My dad] He used to tell me, he was going to kill me, for being the way I am. That he would throw me into the water and nobody would know where I was” – Interview participant, 15 years old, Pucallpa
6	“I had to escape from there [Pucallpa] and I decided to come here [Lima]. My family did not love me, they did not accept me, even my dad he hit me….I ran away” – Focus group participant, Pucallpa

**Table 2:** Illustrative quotes of experiences and vulnerabilities during migration

Theme	Illustrative quotes
1	“I came here, alone to Pucallpa. I escaped. I had to find a way to paid for my own ticket and I came here knowing no one” – Interview participant, 15 years old, Pucallpa
2	“I left at age 15 falsifying my documents. Where did I want to go? To Lima. I am still trying to get there” – Interview participant, 17 years old, Pucallpa
3	“She [trans friend] told me ‘let’s go to Pucallpa’ she said, I tell her ‘but I do not have any papers.’ ‘Let’s go anyways, I’m going to pay,’ she said to me.” – Interview participant, 16 years old, Pucallpa
4	“Migration for trans people is like a chain. That is, they [elder trans women] they help you get your silicon, pay for your ticket, pay for your luggage, buy everything you need, but once you arrive to Lima they charge you. You work on the avenue [street-based sex-work] to pay the, that is how the movement is.”– Interview participant, 20 years old, Pucallpa
5	“They [elder trans women] tell you that prostitution gives money there [Lima], they encourage you, they encourage you, they compare what you gain here to what you can have there. They come to your house, or they find you in a nightclub or they go to the square… They say “do you dare? I can pay your ticket”. They tell you, you can stay at their apartment, they’ll show you Lima, you will work together on the street, and you will only have to pay them little by little. There are many who accept, the other year I know seven that left.”– Focus Group Participant, Iquitos
6	“‘ ‘Let’s go to Lima’ she [elder trans woman] said to me, ‘in Lima, we charge 50 to 100 soles, you can make your money. I pay for your ticket, and when you work you give it back to me. That’s how we [trans women] go to Lima.” – Interview participant, 28 years old, Iquitos
7	“ I met her [trans Mother] when I went to eat at the restaurant she said ‘why are you coming alone?’ I said, ‘ah, I do not have family here’ she said ‘do you want to work?’ I said, ‘I want to work but I cannot find work,’ she gave me work. She paid me 20 soles a day. I went in at seven in the morning to nine thirty in the evening, I worked all day cleaning rooms, tending the beds and helping in the kitchen washing the dishes.” – Interview participant, 15 years old, Pucallpa

**Table 3:** Illustrative quotes of experiences and vulnerabilities post-migration and during relocation in Lima

Theme	Illustrative quotes
1	“Once I arrived [in Iquitos] I thought I would go to Lima. But, I met a trans friend who told me ‘I am going to take you to the square, I’ll make you pass as my daughter’. She was older than me and she taught me how to make a life here.”–Interview participant, 28 years old, Iquitos
2	“It was hard to stand on the street [engage in street-based sex work] when I got here, but she [elder trans women] talked with them [other trans sex workers], they had a meeting and now they accept me.”– Interview participant, 15 years old, Pucallpa
3	“… I regretted coming to here until one day I met another trans women, and she helped me find my medicine. She helped me so much. From there, she introduced to me other trans women, they helped me work…they are good to me.” – Interview participant, 16 years old, Pucallpa
4	“Oh, the first days I had no experience. I remember a boy came up an asked me “how much do you charge?“ I did not know what to say. I have no money, need to send money home, and need to pay to live. – Interview participant, 22 years old, Lima
5	“Now that I am in Lima, I have to pay to work [sex work]. That is because I’m not complete, well, I do not have an ass, I do not have breasts.” – Interview participant, 16 years old, Lima
6	“There is competition here [Lima]. They do not want us [younger trans women], they do not want you to stand next to them on the street” – Interview participant, 22 years old, Lima
7	“The girls who are under 15 or 16, the ones who just got here, who are just beginning the night life [sex work] and life teaches you. We all went through that. But who will listen to you? The police hit and then the clients hit you… in front of your house, and in the same plaza.” – Interview participant, 28 years old, Iquitos
8	“He [client] grabbed my hair and pushed me, he told me ‘the others, who knows, if a worse one comes, someone uglier than me, he will break your face’”– Interview participant, 22 years old, Lima

### Pre-migration/place of origin (Peruvian Amazon)

The pre-migration stage was commonly characterized by seeking to relocate due to poverty, transphobia, and violence. When discussing motivations for seeking to migrate, several FG participants noted economic precarity and limited opportunities for trans women from rural villages (Table 1 (T1) Quotes 1–3 (Q1-3)). Participants also described their experiences with family as relationships characterized by neglect. For example, one participant’s mother abandoned her for increasing lengths of time beginning at age 6 (T1 Q3) For some, rejection was acutely connected with transphobia (T1 Q4) and, for some, led to threats of murder and rape (T1Q5,6).

Due to the intersecting factors of familial poverty, childhood physical and sexual abuse, and familial rejection of identity, several trans adolescents described their decision to leave their families as an “escape” (T1 Q6). During childhood, multiple participants reported experiencing physical violence, sexual violence, and rejection of gender identity by a family member. One participant reported being threatened with murder by her father, who threatened that nobody would be able to find her body (T1 Q5). An elder trans woman who had migrated from a smaller village in the Amazonian region of Peru to Iquitos described many younger trans women “escaping” Amazonian villages due to physical violence, sexual violence, and threats (T1 Q6). Participants described these experiences of violence and rejection as key motivating factors for migration.

It is important to note that particularly since many participants were both trans and young, they were reliant on family members for care and financial support, but simultaneously experienced violence perpetuated by family members. In this context, participants underscored that migration was also considered a form of displacement due to pervasive experiences of transphobic violence. These factors jointly constituted the desire and motivation to leave and migrate to another location. For example, some participants illustrated how many people are motivated to migrate to seek jobs and educational opportunities, but that for young trans women, the main motivation is to escape violence. Moving to a city like Pucallpa, Iquitos, or Lima was perceived as an opportunity to search for better living conditions, to escape intergenerational poverty, family instability, and neglect, as well as the very real threat of violence.

### Migration/travel, in transit

Most participants described deciding to leave and “escaping” their villages long before they were able to finance their migration. As trans women often left their homes in times of crisis or as minors, they frequently did not have identification and most described starting their migration process alone (T2 Q1). Some participants falsified documents (T2 Q2) to travel, and others sought support from elder trans women to obtain needed documentation to continue their journey (T2 Q3-4).

Participants frequently described Lima as their desired destination (T2 Q2, Q5) and some noted interest in traveling to other countries, namely the Unites States, Italy, and/or Brazil. Financial resources were described by all participants as a main barrier not only to initiating migration but through the migration process. Elder trans women were described as central to facilitating intranational migration (T2 Q3-7). While for some, elder trans women were described offering acts of support, most noted exploited labor especially as related to initiation or engagement in sex work (T2 Q4-5). Participants described being approached by a variety of people including police, clients, and other trans women while in public spaces, being identified as an isolated and vulnerable individual, and then offered work. The jobs they were offered included cleaning, housekeeping, and preparing food (T2 Q5-6). Due to lack of other employment opportunities, participants were dependent on these adult figures for safety and stability, while working over ten hours a day.

### Post-migration/destination

As participants recounted their experiences with mobility, the distinction between during and post-migration trajectories was often blurry as the destination city could shift due to resource availability and/or the process could take more time than anticipated (T2 Q2; T3 Q1). Some younger participants spoke of meeting other trans women, creating bonds over shared experiences, and finding community as a key source of support for life in a new city (T3 Q1-3). For example, to “stand in the street,” language often referenced by participants when talking about sex work, most noted the need to advocate to work on a specific block or location. Therefore, elder trans women, often described as trans mothers, were central to negotiations of block approval (T3 Q2). Many also echoed sentiments that without this introduction and approval, they would have been subjected to within-community violence.

Paralleling experiences during migration, trans mothers were described as taking on a caregiver role for young trans women, often calling them their “daughters” (T3 Q1). Yet, participants also spoke of their relationship with their trans mothers as complicated due to financial entanglements. For some, trans mothers loaned them money to get gender-affirming medical care in Lima, which needed to be paid back slowly. Some also noted the pressure to continue using feminizing procedures until they were “complete” to compete with other trans women and to attract more clients (T3 Q4). This pressure was described as leading some trans women to take on more loans, with sex work rapidly becoming the only perceivable way to pay back their debts. Others described how young trans women who were seen as especially beautiful received more significant loans to travel to other cities in Europe and Argentina for gender-affirming medical care. Other participants noted that given the pressures from clients perceptions of youth and beauty fostered competition among trans women who engaged in sex work (T3 Q6).

Participants detailed how the violent experiences pre- and during migration continued once they arrive to their anticipated destination city–for most, Lima. Many participants spoke of their introduction to sex work as painful and frightening, explaining that they did not earn much money and were unsure what to charge (T3 Q2). Despite working long and hard hours, many reported being unable to support themselves economically in a larger city. Some also needed to send money back home to their families of origin, which was an added financial burden. They also mentioned the risk of being caught by the police, and concerns about physical and sexual violence perpetrated by clients (T3 Q6-7).

### Traversing the pre-, during and post-migration process: intersectional stigma

Oppression due to interactional and enacted forms of stigma were a common thread throughout the intranational migration process as the result of the confluence of social class, gender identity, age, mobility, and violence. As described throughout the interviews and FGs, participants were vulnerable to multi-level violence; first by family members pre-migration and then by clients, law enforcement, and, at times, from other trans women, during and post-migration. Overall, many noted how, besides also acting as role models and giving support, elder trans women can also replicate cycles of violence and oppression towards the younger generations, underscoring the complexity of intergenerational trauma. Participants described how violence was an ongoing aspect of their lives throughout migration trajectories and shadowed experiences of relocation, including limited protection by law enforcement (T3 Q7). Post-migration, descriptions of violence were frequently tied to sex work and linked to concerns about sexual health. Due to their youthfulness, younger trans women were often perceived by elder trans women as competition for clients, at times resulting in physical violence perpetuated by elder trans women (T2 Q2-3), particularly when elder woman received less clients than the younger woman. Trans women also experienced violence from clients, including being grabbed, pushed, and threatened by a client (T3 Q7-8). Rooted in intersectional stigma at the micro, meso, and macro levels, experiences of oppression and exploitation, characterized current and recalled migratory trajectories of adolescent and adult trans women participants.

## Discussion

This study advances understandings of intranational migration and forced displacement as key determinants of trans women’s health by evidencing how dimensions of violence at the intersection of classism, ageism, and cisgenderism render trans women highly vulnerable at every step of their migratory journeys. Experiences of intranational mobility and relocation were described as uniquely tied to intersectional transphobic stigma and violence, which impact and can magnify health vulnerabilities in adolescence and beyond for trans women in Peru who migrated intranationally. To attend to the complexities of intranational migration, our approach leveraged qualitative methodology at three phases to understand the arc of the migration experience. In doing so, our findings detailed how the pre-migration stage was characterized by poverty, transphobia, and violence frequently motivating the decision to migrate in efforts to “escape”. Yet, these forms of oppression and exploitation were also described as pervasive and often replicated during migration and in relocation processes.

Migration has been evidenced as an important determinant of health [1, 6], and for trans women’s health, this study underscores the urgency of an intersectional examination that moves beyond individual health behaviors to include the broader social, economic, and political forces that shape migration and associated health vulnerabilities. In this way, intersectional stigma should not be leveraged to flatten or obsure co-occuring systems of oppression. Rather it can be used to further understand the ways in which unique experiences and the complex forms of violence faced by trans women who belong to multiple marginalized groups impact migration trajectories. Participants narrated key points of intersection that yielded exploitation including age, gender identity, and class. Paralleling other literature [2, 5, 27, 37], trans mothers were described as a support system to mitigate these factors for some participants. However, cross-generational and within-trans community supports were complicated by financial and social power dynamics as well as community-level cultural expectations and social norms. With an eye to the complexity of community dynamics to both mitigate and foster health vulnerabilities for trans women migrants, further research is needed to examine how social networks impact processes of belonging and access to material and social support during mobility and resettlement especially across the life course.

Precarious and exploitative labour in and around erotic economics (e.g., survival sex, sex work, transactional sex practices) can impact the structure of pathways for transnational migration, with evidence examining the experience of Latin American trans women [38, 39]. This literature has shown how erotic labour takes on many integral dimensions within migration trajectories, especially from Latin America to the US and to Europe. For some, erotic labour provides autonomy (e.g., economic, pleasure) and linkages to existing trans communities in receiving countries [40]. Extending these results to intranational migration, our study highlights the importance of acknowledging erotic economics in structuring experiences of intranational migration for trans women. Sex work is legal in Peru, however transphobia and transmisogyny were commonly described by participants as heightening violence faced while engaging in street-based sex work. By presenting findings through an intersectional lens, we seek to place erotic labour within the larger sociopolitical forces that constrain trans people’s human rights (e.g. lack of gender identity legislation, ongoing violence’s perpetrated by law enforcement) and result in social and economic marginalization.

Characteristics of migration, displacement, and mobility were frequently described in a way that underscored movement as an act of resistance and an enactment of agency to seek gender-affirming spaces and resources outside of hometowns and families. Participants described transphobic stigma and violence that motivated relocation as linked to the intersection of social class and gender identity. This finding is also reported in other trans health literature [2, 41]. Our findings highlight that for trans adolescents and women, greater attention is needed to how violence is perpetrated at the hands of those who are in positions to protect them and care for them (e.g. caregivers, police). This violence may start with family members pre-migration, and then may include police and even at times trans mothers during- and post-migration. These narratives of intersectional stigma and violence underscore that the lack of protections for trans women at the institutional/structural level which can increase vulnerability to violence during- and post-migration trajectories [42–44].

This study did not focus on HIV prevention and care, however, given the frequency of narratives that detailed sex work, migration, and violence, it is important to emphasize that HIV vulnerabilities are linked to intersectional stigma. Young trans adolescents and women ages 16–24 years are a critical group for primary HIV prevention efforts in Peru, given sharp increases in HIV prevalence among trans women ages 25–29 years [28, 45]. Research has evidenced several risk factors associated with HIV infection in trans women, namely STI co-infections and condomless receptive anal sex [29, 31, 46, 47]. Further, HIV vulnerabilities for trans women occur within the context of widespread violence in Peru, including social exclusion and intersectional stigma in school and employment leading to sex work and internalization of transphobic mistreatment. To advance the Peruvian literature on HIV vulnerability, future assessments should include intranational migration as a key determinant of health to better understand the dynamics of this epidemic. International and local policies should prioritize trans women who have migrated as a priority for health and social services.

These findings should be understood as a first step and interpreted alongside several limitations. The study was conducted prior to the COVID-19 pandemic, which in Peru and in the Latin American region has severely impacted the lives of trans women [26]. Further, this study assessed the arc of intranational migration for Peruvian trans adolescents and women and did not account for other significant dynamics related to the impacts of the Venezuelan diaspora in Peru, the second largest recipient country of displaced Venezuelans. Scholars seeking to further this line of research inquiry should also query the impact of COVID-19 on Venezuelan trans migrants in Peru to provide a more nuanced assessment of the relationship between social networks, gender identity, and migration among trans adolescents and women. Further, this is a small qualitative study and further research with Peruvian trans women who have migrated, especially among young trans women, is needed to better detail pathways and mechanisms that can result in detrimental health outcomes. Despite these limitations, our results provide important evidence from the Peruvian context regarding intranational migration trajectories illustrating that intersectional transphobic stigma and violence dominate motivations for and experiences of mobility and relocation for trans women.

## Conclusion

Migration, including pre-, during- and post-migration trajectories, is an important health determinant for Peruvian trans women and associated with health vulnerabilities. Experiences of migration, often initiated in adolescence, were described as tied to intersectional stigma and transphobic violence. Further research is needed with this population to determine needs and supports that would address the inequities and intersecting harms faced before, during, and after migration for trans women. Trans-specific health and social services prioritizing youth are vital, as are youth services that prioritize trans-specific competency and gender-affirming needs. Holistic programs and interventions that include gender-affirming care, sexual health care, and housing and employment will be necessary to protect trans women migrants and offset potential health-harms of migration. Shelters for intranational trans migrants where they will not be exploited are direly needed, and ideally where they may be linked to educational opportunities to obtain basic education, and health services to mitigate pathways (re)producing health inequities.

## Data Availability

The data that support the findings of this study are available from the corresponding author, APB, upon reasonable request.
